# Brain Regulation of Cardiac Function during Hypoglycemia

**DOI:** 10.3390/metabo13101089

**Published:** 2023-10-18

**Authors:** Matthew E. Chambers, Emily H. Nuibe, Candace M. Reno-Bernstein

**Affiliations:** Division of Endocrinology, Metabolism, and Diabetes, University of Utah School of Medicine, Salt Lake City, UT 84112, USAu1284157@utah.edu (E.H.N.)

**Keywords:** hypoglycemia, cardiac arrhythmias, sympathetic nervous system, parasympathetic nervous system, brain, diabetes

## Abstract

Hypoglycemia occurs frequently in people with type 1 and type 2 diabetes. Hypoglycemia activates the counter-regulatory response. Besides peripheral glucose sensors located in the pancreas, mouth, gastrointestinal tract, portal vein, and carotid body, many brain regions also contain glucose-sensing neurons that detect this fall in glucose. The autonomic nervous system innervates the heart, and during hypoglycemia, can cause many changes. Clinical and animal studies have revealed changes in electrocardiograms during hypoglycemia. Cardiac repolarization defects (QTc prolongation) occur during moderate levels of hypoglycemia. When hypoglycemia is severe, it can be fatal. Cardiac arrhythmias are thought to be the major mediator of sudden death due to severe hypoglycemia. Both the sympathetic and parasympathetic nervous systems of the brain have been implicated in regulating these arrhythmias. Besides cardiac arrhythmias, hypoglycemia can have profound changes in the heart and most of these changes are exacerbated in the setting of diabetes. A better understanding of how the brain regulates cardiac changes during hypoglycemia will allow for better therapeutic intervention to prevent cardiovascular death associated with hypoglycemia in people with diabetes. The aim of this paper is to provide a narrative review of what is known in the field regarding how the brain regulates the heart during hypoglycemia.

## 1. Introduction

Hypoglycemia is very common in people with diabetes, especially those with insulin-treated diabetes. The frequency of hypoglycemia in people with type 1 diabetes is about two episodes per week, with at least one severe episode per year [1]. Severe hypoglycemia in people with type 2 diabetes occurs in about 3–25% of individuals; however, this number is much higher in people with insulin-treated type 2 diabetes [2,3]. A recent study revealed that more than 80% of people with type 1 diabetes and almost 50% of those with type 2 diabetes experienced hypoglycemia during a one-month period, with 14% and 9%, respectively, experiencing severe hypoglycemia [4]. 

Clinically, hypoglycemia can be divided into three levels [5]. Level 1 is defined as glucose levels being between 54 and 70 mg/dL. Level 2 is defined as plasma glucose being below 54 mg/dL. Level 3 hypoglycemia is defined as a very serious event that requires assistance from another person to return glucose concentrations to a normal range. This can be as little as someone handing the person orange juice or taking a trip to the emergency room.

### 1.1. Impaired Awareness of Hypoglycemia

**Figure 1:**
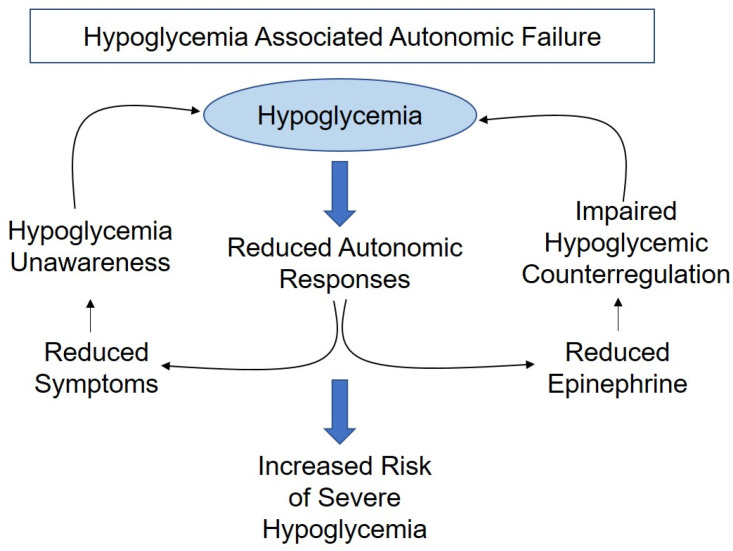
Hypoglycemia-associated autonomic failure.

In many people with insulin-treated diabetes, an impaired awareness of hypoglycemia may occur. Repeated episodes of hypoglycemia can lead to hypoglycemia-associated autonomic failure (HAAF) (Figure 1) [6]. HAAF can alter the brain pathways involved in detecting and responding to hypoglycemia [7,8]. This can lower the glucose threshold required to initiate the counterregulatory response (CRR) to well below 70 mg/dL. This can be very dangerous, as it could lead to more severe hypoglycemic episodes if glucose levels are not corrected quickly. 

### 1.2. Severe Hypoglycemia

Intensive glycemic control, as is indicated by a targeted A1C of less than 7% or as close as is reasonably possible to glucose levels in people without diabetes [9], in diabetes markedly increases the risk for severe hypoglycemia, which can be fatal [10,11,12,13,14]. Hypoglycemia accounts for 6–10% of all deaths in people with type 1 diabetes [15,16,17]. Sudden unexplained death occurs in people with type 1 diabetes, with the death rate being 10-fold higher in young people with type 1 diabetes than in non-diabetics [18]. This ‘dead-in-bed’ syndrome identifies otherwise healthy young individuals with type 1 diabetes who were found dead in bed with no clear cause of death [19], accounting for up to 27% of all unexplained deaths in people with type 1 diabetes [20,21]. Case reports suggest that severe hypoglycemia due to excess insulin administration contributed to these deaths [22,23]. Glucose levels of 10 mg/dL have been reported in a case report of such an individual who died from the dead-in-bed syndrome [22]. Cardiac autonomic dysfunction is highly associated with mortality in diabetes and is perhaps the reason for the increased risk of death associated with severe hypoglycemia [24]. Cardiac autonomic dysfunction increases mortality from arrhythmias and myocardial infarction in people with diabetes [24]. Besides sudden death from severe hypoglycemia, the risk of cardiovascular events and mortality in people who experience severe hypoglycemia is increased 1.5–6-fold [25]. 

## 2. Methods

PubMed and Google Scholar were used to search for relevant articles for this review. Search strings included: “brain and cardiac arrhythmias”, “sympathetic nervous system and cardiac arrhythmias”, “parasympathetic nervous system and cardiac arrhythmias”, “brain and hypoglycemia”, “brain, hypoglycemia, and cardiac arrhythmias”, “brain and cardiac function”, “brain, hypoglycemia, and cardiac function”, and “brain and heart”.

## 3. Hypoglycemia and the Counterregulatory Response

Both the sympathetic (SNS) and parasympathetic (PNS) nervous systems play a role in regulating blood glucose. When glucose levels drop, the body responds with the hypoglycemic CRR. This normally includes increasing epinephrine, norepinephrine, cortisol, growth hormone, and glucagon levels; increasing hepatic glucose production; and decreasing insulin secretion and glucose uptake in peripheral tissues [26]. People with type 1 diabetes often have an absent glucagon response, making them heavily reliant on the epinephrine response to increase glucose levels. Clinically, glucose levels below 70 mg/dL initiate neuroendocrine responses to hypoglycemia in people without diabetes, whereas people with type 1 diabetes and hypoglycemia unawareness might have this response occur at a lower glucose threshold, partly due to the absent glucagon response [7,27]. 

**Figure 2:**
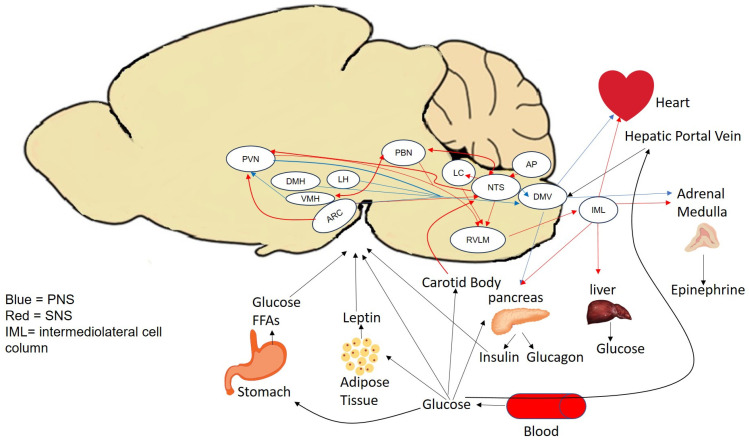
Glucose-sensing pathways. Red arrows represent the SNS. Blue arrows represent the PNS. (Figure 2 is not meant to contain every single afferent and efferent pathway from all brain regions; it is meant to give an overview of the main pathways).

The brain is involved in the control of whole-body homeostasis (Figure 2). The brain maintains a balance of the secretion of both orexigenic and anorexigenic peptides that respond to glucose levels and other metabolic signals [28,29]. The hypothalamus is the main center of homeostatic control, particularly due to its location near the third ventricle, which allows access to different circulating peptides and hormones such as leptin, insulin, glucagon, polypeptide YY, and cholecystokinin [29]. Insulin is known to cross the blood–brain barrier [30]. However, in most studies, it is hypoglycemia and not necessarily the hyperinsulinemia that results in changes to the heart [31,32]. 

There are regions in the brain that contain glucose-sensing neurons. Additionally, there are glucose-sensing cells located in the pancreas, mouth, gastrointestinal tract, portal vein, and carotid body [33]. The peripheral glucose sensors send signals via afferent pathways to different regions of the brain (Figure 2). Within the brain, there are multiple areas that are able to detect hypoglycemia. These regions include the ventromedial hypothalamus (VMH), arcuate nucleus (ARC), lateral hypothalamus (LH), paraventricular nucleus of the hypothalamus (PVN), medial and lateral arcuate nucleus (ARC), dorsomedial hypothalamus (DMH), area postrema (AP), parabrachial nucleus (PBN), nucleus tractus solitarius (NTS), and dorsal motor nucleus of the vagus (DMV) [34,35,36,37,38,39,40,41]. Specifically, in the VMH, hypoglycemia activates the glucoregulatory neurocircuits [42,43,44]. 

Among the various types of neurons found within the brain are glucose-excited (GE) and glucose-inhibited (GI) neurons which are important for regulating blood glucose levels. In general, GE neurons increase neuronal activity in response to glucose, while GI neurons decrease neuronal activity in response to glucose [45,46]. Thus, GI neurons are activated during hypoglycemia and GE neurons might be less activated. The major region that contains GE and GI neurons is the hypothalamus, and within the hypothalamus, the VMH is the most studied brain region for glucose sensing and hypoglycemia. Decreased glucose entry into the neurons of the hypothalamus leads to increased ratios of AMP/ATP, the activation of AMPK, and finally the formation of nitric oxide, which acts as a neurotransmitter [47,48]. This leads to the CRR during hypoglycemia, which includes secretion of norepinephrine and epinephrine [49]. 

Other regions involved in glucose sensing are in the brainstem [50]. Clinically, the brainstem activation during hypoglycemia in people with type 1 diabetes and healthy controls has been observed [51]. Within the brainstem, the NTS, the area postrema, the dorsal motor nucleus of the vagus (DMV), and the subfornical region have been shown to contain glucose-sensing neurons [52,53,54]. The area postrema is located at the base of the hindbrain [35]. Because of where this circumventricular organ is located, it is able to rapidly transduce glucose-sensing information from the periphery. Additionally, the median eminence, located on the floor of the mediobasal hypothalamus, is important for glucose sensing because it is located adjacent to the third ventricle without a proper blood–brain barrier, allowing this region to detect hormones and nutrients in the blood [55,56,57]. During hypoglycemia, glucose-sensing neurons within the NTS send signals via GABAergic projections to the DMV, leading to the CRR [58,59]. The DMV also projects to the PBN and cerebral cortex [60]. A study of healthy humans found that electrical stimulation of the antero-lateral surface of the neck, via the vagus nerve along the carotid artery led to the activation and deactivation of several brain locations including the NTS, PBN, thalamus, caudate, hippocampus, and spinal trigeminal nucleus, suggesting that hindbrain activation can project anteriorly to other regions in the brain [61]. Additionally, within the NTS are both noradrenergic (A2) and adrenergic (C2) neurons that project to the PVN to release corticotrophin-releasing hormones during hypoglycemia [62]. 

## 4. Brain Connection to the Heart

Many brain regions connect to peripheral organs, such as the heart, to control body functions. Some of these brain regions that connect directly to the heart include the anterior cingulate cortex, amygdala, parabrachial nucleus, hypothalamus, periaqueductal grey matter, anterior insula, and regions of the medulla [63].

The stellate ganglia and vagus nerve connect directly to the sinoatrial (SA) node of the heart to control heart rate. The NTS contains GABAergic neurons that exhibit tonic inhibitory control. The frontal cortex also has links to the effects of both the SNS and PNS on the heart [64]. Specifically, prefrontal cortical activity inhibits brainstem cardioacceleratory circuits [65]. Clinically, the medial prefrontal cortex is activated during hypoglycemia, which is additionally reduced in people with hypoglycemia unawareness [66,67].

The activation of the amygdala has been implicated in increased heart rate and decreased heart rate variability. SNS neurons can be activated in the rostral ventrolateral medulla (RVLM), either directly or via the decreased inhibition of neurons in the caudal ventrolateral medulla (CVLM), which leads to increased SNS activity [68]. Decreased PNS activity can occur through the inhibition of NTS neurons, leading to the inhibition of neurons in the active nucleus ambiguous (NA) and dorsal vagal motor nucleus (DVN). 

The PVN, LH, and VMH are important for hypoglycemia-induced catecholamine release [69,70]. Within the LH, hypoglycemia leads to orexin secretion, which stimulates C1 neurons within the RVLM. From the RVLM, C1 and glutamatergic neurons innervate the adrenal gland, which can lead to epinephrine and glucagon secretion [71]. Stimulating the LH can alter the electrocardiogram (ECG) by depressing ST segments and increasing heart rate, while stimulating the anterior hypothalamus results in PNS activity and bradycardia [72].

The parvicellular neurons in the PVN initiate the SNS response via pre-ganglionic spinal efferents [73,74]. Parasympathetic (vagal) innervation is relayed from the PVN to the DVN, which then relays it to the peripheral organs. The PVN’s innervation of the DVN regulates the vagal parasympathetic efferent neurons and directly activates the sympathetic nerves in the spinal cord.

## 5. Sympathetic vs. Parasympathetic

**Figure 3:**
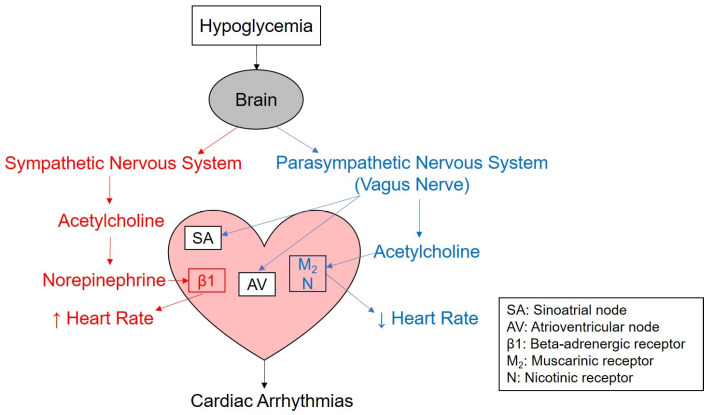
The autonomic nervous system regulation of hypoglycemia-induced cardiac arrhythmias.

The main role of the SNS is to increase heart rate, while the PNS decreases heart rate. Both systems consist of pre- and post-ganglionic neurons with acetylcholine (ACh) as the neurotransmitter acting between these neurons (Figure 3). The cardiac sympathetic pre-ganglionic nerves originate from the T1–T4 regions of the thoracic segments of the spinal cord. The post-ganglionic neurons of the SNS secrete norepinephrine. 

The SNS receptors in the heart are β1, β2, α1, and α2 adrenergic receptors. α1 and β1 are responsible for vasoconstriction, whereas β2 is responsible for vasodilation [75]. The activation of β1 receptors leads to increased heart rate, increased contractility, and increased atrioventricular node conduction. The release of epinephrine from the adrenal medulla can also circulate to the heart and act on local adrenergic receptors.

The PNS neurons stem from the mid-brain, pons and medulla oblongata [75]. The vagus nerve carries most of the PNS’s signals from the brain to the heart. The vagus nerve directly innervates the SA and AV nodes. The post-ganglionic neurons are cholinergic and bind to either nicotinic or muscarinic (M_2_ or M_3_) receptors. The nicotinic receptors are located between pre- and post-ganglionic neurons of both the SNS and PNS.

M_2_ receptors are located within the nodal cells and atrial tissue of the heart but not in the ventricles [75]. Activation of the M_2_ receptors slows the heart rate down by decreasing conduction velocity through the AV nodal cells and reducing depolarization. It also reduces contractility, leading to a decreased cardiac output. 

The nicotinic receptors are located in various tissues and have several subtypes, mainly divided into muscle-type (peripheral) and neuronal-type. In the brain, most mammalian subtypes are α4β2 or α7 [76,77]. The neuronal type of receptor is located in most post-ganglionic neurons [78]. Nicotinic receptors are additionally located within the adrenal medulla, and upon activation lead to epinephrine secretion [79]. 

The SNS and PNS each act antagonistically on the heart to control its function. Many studies have found that both the SNS and PNS influence the heart during hypoglycemic events (Figure 1). Heart rate variability (HRV) is a measurement of SNS and PNS activity. HRV is expected to decrease in most cases of hypoglycemia as the SNS is activated. A recent study found that in children with type 1 diabetes, HRV parameters changed during spontaneous nocturnal hypoglycemia even before the hypoglycemia was detected on a continuous glucose monitor [80]. This indicates that autonomic modulation occurs even prior to being able to detect hypoglycemia. 

Clinical studies have shown that hypoglycemia experienced in the daytime vs. the nighttime has contrasting autonomic effects on the heart [81,82]. For instance, during the day, hypoglycemia elicits tachycardia, while at night, hypoglycemia elicits bradycardia. The prevalence rate of nocturnal hypoglycemia in young people with type 1 diabetes is 68% [83,84]. Furthermore, in sleeping individuals with type 1 diabetes, the PNS has been shown to regulate hypoglycemia-induced cardiac rhythms [82]. Excess vagal tone has also been shown to trigger cardiac arrhythmias [85]. 

At night, sympathetic withdrawal and parasympathetic activation lead to bradycardia and ventricular ectopic beats [81,82]. Furthermore, at night, people with diabetes may have longer episodes of hypoglycemia due to not waking up from the event. This could account for instances of the ’dead-in-bed’ syndrome. Animal studies also show that prolonged hypoglycemia decreases heart rate and leads to atrioventricular (AV) heart block, which precedes mortality [86,87,88]. Furthermore, blocking the SNS and PNS separately in rats was able to prevent fatal heart block during severe hypoglycemia [86,87]. Taken together, the increased risk of hypoglycemia-induced mortality may be due to SNS withdrawal and/or PNS over-stimulation, but this is just speculation as no studies have directly confirmed this. This mounting evidence indicates that the PNS is involved in hypoglycemia-induced arrhythmias. While increasing the PNS tone has been thought to be protective against cardiovascular events such as myocardial infarction and heart failure, the role of the PNS in mediating cardiac arrhythmias during hypoglycemia is less clear.

## 6. Hypoglycemia-Induced Cardiac Arrhythmias

Through several clinical trials, hypoglycemia has been associated with death or adverse cardiac outcomes. However, there is limited clinical data on how/if severe hypoglycemia leads to fatal cardiac arrhythmias due to the nature of not knowing when a severe hypoglycemic episode will occur. However, clinical studies of moderate levels of hypoglycemia or spontaneous hypoglycemic events have provided some insight into how hypoglycemia and possibly severe hypoglycemia, might lead to cardiac abnormalities. Clinical studies using ECGs have found that cardiac arrhythmias associated with cardiac cycle (QTc) prolongation occur during moderate hypoglycemia in type 1 diabetes [31,89,90,91,92], with more severe hypoglycemia associated with a greater prolongation [93]. QT prolongation can predict cardiovascular mortality and all-cause mortality in people with diabetes [94,95]. Additionally, clinical studies have revealed that bradycardia, atrial fibrillation, ventricular ectopic beats, and ventricular tachycardia can occur during hypoglycemia [96]. ECG alterations have also been found in people with type 1 diabetes and healthy subjects during hypoglycemia [82,97,98]. These alterations in ECG readings during hypoglycemia have been observed in atrioventricular conduction, ventricular depolarization, ventricular repolarization, heart rate, and heart rate variability, which may be factors leading to arrhythmias [81,97,99].

Hypoglycemia is not only predominant in people with type 1 diabetes. Hypoglycemia is also associated with an increased risk of cardiac arrhythmias in people with type 2 diabetes and those with a history of cardiovascular disease or at high cardiovascular risk [81]. Ventricular premature beats can occur during hypoglycemia in healthy controls and people with type 2 diabetes [100]. Cardiac arrhythmias are more associated with nighttime hypoglycemia than daytime hypoglycemia. Another study found that nighttime hypoglycemia in insulin-treated people with type 2 diabetes led to atrial fibrillation, bradycardia, and non-sustained ventricular tachycardia [101].

**Figure 4:**
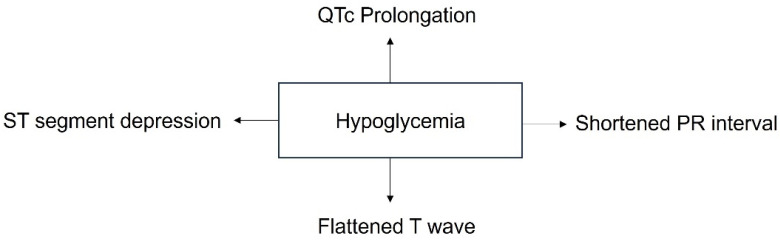
The effects of hypoglycemia on the cardiac electrocardiogram.

In healthy individuals, hypoglycemia decreases the ST segment, flattens the T-wave, shortens the PR interval, and prolongs the QTc interval [97] (Figure 4). In this study, a change in R-wave amplitude was associated with plasma norepinephrine and the flattening of the T-wave was correlated with the plasma epinephrine levels, indicating that the autonomic nervous system can alter ECG parameters during hypoglycemia. Together, these data indicate that hypoglycemia can affect atrioventricular conduction, ventricular depolarization, and ventricular repolarization.

In an animal model, insulin-induced severe hypoglycemia led to QTc prolongation and multiple types of cardiac arrhythmias with high-degree atrioventricular block, often preceding mortality [87]. Third-degree heart block was highly sensitive and specific at predicting mortality during severe hypoglycemia in these studies [87,88]. However, during more moderate levels of hypoglycemia in this animal model, cardiac arrhythmias were rarely observed. This paradoxical decrease in heart rate, in addition to QTc prolongation and ventricular arrhythmias, during hypoglycemia is consistent with what is observed clinically [81,89,90,91,102,103,104].

These many clinical and animal studies reveal that hypoglycemia and the subsequent physiological CRR are correlated with an increased risk in cardiac arrhythmias. However, the mechanisms causing these arrhythmias are not well understood. It has been suggested that sympathoadrenal activation due to brain neuroglycopenia is involved.

### 6.1. Sympathetic Nervous System and Cardiac Arrhythmias

Clinical and animal studies together point to epinephrine or the SNS response as major contributors to hypoglycemia-induced cardiac arrhythmias or abnormalities. Clinically, QT prolongation during hypoglycemia could be reduced using selective beta blockades, leading to the conclusion that the main cause of QT prolongation during hypoglycemia is sympathoadrenal stimulation [31]. However, in animal studies, although QT prolongation occurs, it does not seem to be associated with hypoglycemia-induced cardiac arrhythmias [88].

An experiment in a rat model that supports these clinical data showed that fatal cardiac arrhythmias during severe hypoglycemia could be prevented by a nonselective β-adrenergic blockade [87]. An additional study in rats showed that the infusion of a β_1_-blocker during severe hypoglycemia prevented nearly all types of cardiac arrhythmias [105]. Moreover, brain ICV glucose infusion during a severe hypoglycemic clamp in rats reduced the amount of fatal heart block, which was associated with a decrease in plasma norepinephrine [87]. This indicates that the brain is a direct mediator of hypoglycemia-induced cardiac arrhythmias. 

To further support these findings, recurrent hypoglycemia protected rats from fatal cardiac arrhythmias during a subsequent severe hypoglycemic episode [88]. This decrease in cardiac arrhythmias was associated with a blunted epinephrine response during severe hypoglycemia, again pointing to the major role of epinephrine in mediating hypoglycemia-induced cardiac arrhythmias.

However, a contradictory study has shown the limited impact of the SNS in severe hypoglycemia-induced cardiac arrhythmias. In the experiment, both chemical sympathectomy and adrenal demedullation in rats had no significant effect on arrhythmias compared with the controls [86]. Another study also found that adrenalectomy in cats did not protect them from ECG changes induced by stimulating the mesencephalic reticular formation, suggesting that the effects on the ECG were due to intracardiac SNS activity and not the circulating epinephrine [106]. Alternatively, a rat study found that the activity of the PNS through ACh activation of nicotinic receptors is the primary mediator of severe hypoglycemia-induced fatal cardiac arrhythmias [86].

### 6.2. Parasympathetic Nervous System and Cardiac Arrhythmias

It seems counter-intuitive that the PNS would increase cardiac arrhythmias during hypoglycemia. However, many recent clinical studies have found that hypoglycemia occurring at night is more associated with cardiac arrhythmias and bradycardia, compared to hypoglycemia experienced in the daytime [82]. Animal studies have shown that surgical vagotomy and nicotinic receptor blockade in non-diabetic rats reduced fatal cardiac arrhythmias during severe hypoglycemia [86]. There are limited data to date that show a direct correlation between PNS activation and hypoglycemia-induced cardiac arrhythmias. We can speculate that prolonged hypoglycemia leads to increased PNS stimulation as SNS activity is withdrawn, and that this leads to changes in the ECG and increases the risk and amount of cardiac arrhythmias.

### 6.3. Other Factors Involved in Cardiac Arrhythmias

#### 6.3.1. Potassium

Insulin and epinephrine’s effects on potassium during hypoglycemia can lead to hypokalemia, which is associated with cardiac arrhythmias. Animal studies have shown that potassium supplementation during severe hypoglycemia in non-diabetic and streptozotocin-diabetic rats tended to reduce cardiac arrhythmias [87]. Additionally, the infusion of glibenclamide to block K_ATP_ channels during severe hypoglycemia also reduced fatal cardiac arrhythmias in rats [107]. Furthermore, clinical studies have shown that potassium infusion during hypoglycemia in healthy men reduces QT dispersion [31]. 

#### 6.3.2. Sodium

Sodium glucose transporter 2 (SGLT2) inhibitors area treatment for diabetes that act to increase glucose loss through urine. SGLT2 inhibitors also reduce all-cause mortality and cardiovascular mortality [108]. It is also coming to light that SGLT2 inhibitors reduce sudden cardiac death possibly due to reduced cardiac arrhythmias [109]. One recent paper reports that people with diabetes and SGLT2 inhibitors had lower atrial fibrillation but not ventricular arrhythmias [110]. Although SGLT2 is not located within the heart, its role in cardiovascular function is either off-target, by reducing SNS activity, reducing prolonged ventricular repolarization, or reducing inflammation and oxidative stress, or the drugs used in these studies might have had a slight inhibitory effect on the SGLT1, which is found in cardiomyocytes [111]. Additionally, SGLT2 inhibitors are known to induce ketoacidosis [109]. An increased production of ketone bodies during hypoglycemia is important for energy utilization [112]. While there is evidence of this association with improved heart failure outcomes [113], there is limited/no evidence of how SGLT2 inhibitors might reduce arrhythmias during hypoglycemia. 

#### 6.3.3. Calcium

Calcium signaling pathways are downstream of both the SNS and PNS. Calcium is important for normal cardiac contraction. Yet, an excess influx of calcium into the cell, as might occur during stress or hypoglycemia, can cause cardiac arrhythmias [114]. One way for cardiac arrhythmias to occur is through “leaky” ryanodine receptors (RYR2) [115]. It is well established that cardiac RYR2 can be activated by upstream beta adrenergic signaling from the SNS [116]. More recently, the PNS regulation of RYR2 has been established, where PNS stimulation leads to changes in phosphorylation of RYR2 at multiple sites which can alter the stability of RYR2 by reducing binding of the co-factor calstabin-2 [117,118]. Furthermore, cardiac-specific RYR2 transgenic mice have bradycardia and arrhythmias [119]. Thus, cardiac calcium signaling may be a common downstream pathway of both the SNS and PNS that can lead to fatal cardiac arrhythmias during severe hypoglycemia.

## 7. Hypoglycemia’s Alteration of Cardiac Function

Hypoglycemia’s effects on the heart are not limited to arrhythmias. Besides cardiac arrhythmias induced by hypoglycemia, there are other consequences of hypoglycemia on the heart. Hemodynamic, prothrombotic, and inflammatory changes, and increased oxidative stress, are a few of the factors that influence the heart during hypoglycemia. Many of these might also be a mechanism leading to cardiac arrhythmias during a hypoglycemic event.

### 7.1. Hemodynamic

SNS activation during hypoglycemia leads to increased heart rate, systolic blood pressure, cardiac output, and ejection fraction, and decreased diastolic blood pressure [120,121]. The increased heart rate is mediated by β1 adrenergic receptors. There is also an increased myocardial contractility and a decreased peripheral arterial resistance [122,123,124]. This leads to increased cardiac output and peripheral systolic blood pressure mediated by α and β2 adrenergic receptors [125]. 

### 7.2. Prothrombotic

During hypoglycemia, the SNS response to increase catecholamines has a direct effect on platelet activation and aggregation. These procoagulant and prothrombotic effects induce platelet aggregation, activation, and secretion. Epinephrine stimulates, and the blockade of α2 adrenergic receptors inhibits, platelet aggregation and activation [126]. Specifically, hypoglycemia increases platelet factor 4 and plasma β thromboglobulin which leads to platelet activation [126].

Clinical studies have shown increased platelet-monocyte aggregation, P-selectin levels, and plasminogen activator inhibitor 1 levels [127,128]. Fibrinogen and factor VIII also increase while platelet count decreases. Part of this increase in factor VIII is due to the release of the von Willebrand factor from endothelial cells during hypoglycemia [129]. In people with type 2 diabetes, hypoglycemia gives rise to prothrombotic changes in fibrinogen and C3 levels, and enhances platelet reactivity [130]. Some of these changes are increased in diabetes and can last up to 7 days after hypoglycemia [130].

### 7.3. Pro-Inflammatory

Hypoglycemia induces an inflammatory response [127]. The number of lymphocytes and monocytes increases in response to hypoglycemia and this can last for up to 7 days in people with type 2 diabetes [131]. Glucose levels during recovery from hypoglycemia can affect the improvement of endothelial dysfunction and inflammation, indicating that the normo- vs. hyperglycemia after hypoglycemia is more beneficial [132]. During acute hypoglycemia, people with type 1 diabetes have an increased CD40 expression on monocytes and plasma sCD40L concentrations and an upregulation of the intracellular adhesion molecule (ICAM), vascular cell adhesion molecule (VCAM), E-selectin, and vascular endothelial growth factor (VEGF) [133]. Inflammatory cytokines are also elevated during hypoglycemia, including interleukin (IL) 6, tumor necrosis factor a, IL-1β, and IL-8.

### 7.4. Oxidative Stress

Hypoglycemia-induced oxidative stress can lead to tissue damage. Mitochondrial reactive oxygen species (ROS) increase in response to hypoglycemia [134]. Clinically, healthy individuals have an increased ROS production during hypoglycemia [135]. In an animal study, recurrent hypoglycemia in diabetic rats increased brain oxidative stress [136]. Prolonged hyperglycemia is also a known contributor of increased ROS [137]. A recent study showed that reducing the oxidative stress in streptozotocin-induced diabetic rats with a vitamin E treatment protected them against fatal cardiac arrhythmias during severe hypoglycemia [138]. Thus, increased ROS due to hyperglycemia or hypoglycemia may be a contributing factor to hypoglycemia-induced cardiac arrhythmias.

## 8. Clamp Studies vs. Spontaneous Hypoglycemia Studies

Most of the hypoglycemia studies in the literature provide data for studies performed in “clamp” settings, while some are in “spontaneous” hypoglycemic conditions. Hyperinsulinemic clamp studies have several advantages: (1) the rate of the fall in glucose is controlled, (2) the glucose level is clamped where needed for the length of time needed, (3) the ECG can be easily monitored in real time, (4) blood samples can be taken to compare counterregulatory responses, and (5) healthy control participants can be included. The main disadvantage of clamp studies is the presence of supraphysiological levels of insulin. Every clamp study has to take into account that hyperinsulinemia may also be a factor in the results obtained. 

The advantage of spontaneous hypoglycemia studies is that people with diabetes can go about their usual days and their normal fluctuations in glucose throughout the day and night can be recorded, along with any changes in the ECG. Also, even though most of these study participants are treated with insulin (or sometimes other glucose lowering agents), the levels of insulin are not as high as those one might obtain during a clamp setting. A couple of its disadvantages are that (1) the study participants have to wear a continuous glucose monitor (CGM) and a Holter monitor to measure the ECG rhythms, which can be a burden, and (2) results will vary from person to person as the length of hypoglycemia and types of ECG rhythm changes will vary greatly from one person to the next. 

### 8.1. Clamp Studies and ECG

Several studies utilizing clamp conditions report data from both hyperinsulinemic euglycemic and hypoglycemic arms of the studies. This helps us understand how many of the effects on the ECG are due to hyperinsulinemia per se. Study protocols vary on whether both euglycemia and hypoglycemia data are reported during the same day or obtained during randomized crossover studies separated weeks apart. In these clamp studies, the high levels of insulin can get into the brain and be one of the effectors of the results.

Robinson et al. [31] performed clamp studies of healthy subjects. Two euglycemic and two hypoglycemic clamp studies were performed 4 weeks apart. Their QTc increased during hypoglycemia but not euglycemia. In this study, pretreatment with a beta blocker also prevented the QTc prolongation during hypoglycemia. This indicates that hyperinsulinemia per se did not lead to QTC prolongation in this study of healthy individuals.

In a study by Laitinen et al. [97], healthy individuals underwent hyperinsulinemic euglycemic (5 mmol/L) and hypoglycemic (3 mmol/L) conditions for 120 min each, back to back. Hyperinsulinemic euglycemia decreased their T-wave amplitude, T-wave area, and ST segment. Hyperinsulinemic hypoglycemia led to a shortening of the PR interval, greater ST depression, flattening of the T-wave, decrease in T-wave area, and QTc prolongation. 

Another study by Chow et al. [32] performed hyperinsulinemic clamps in people with type 2 diabetes and BMI-matched non-diabetic controls with either euglycemic (6 mmol/L) or hypoglycemic (2.5 mmol/L) clamps for 1 h, but induced 4 weeks apart. During hyperinsulinemic euglycemia, QTc intervals increased slightly, but significantly when compared to baseline levels, while the more robust QTc prolongation occurred during hyperinsulinemic hypoglycemia to a similar extent in both groups. T-wave symmetry decreased during both euglycemia and hypoglycemia in the controls. In people with type 2 diabetes, T-wave symmetry decreased more during hypoglycemia than euglycemia.

A recent study by Andreasen et al. [139] performed hyperinsulinemic euglycemic and hypoglycemic clamps in people with type 1 diabetes. These participants underwent two days of clamp experiments in a random order. Euglycemia (5–8 mmol/L) and hypoglycemia (2.5 mmol/L) were performed on the same day, but the recovery from hypoglycemia was either to hyperglycemia (20 mmol/L) or euglycemia (5–8 mmol/L). Heart rate increased overall during hypoglycemia. Of note, two of the participants experienced bradycardia during hypoglycemia. QTc prolongation occurred during hypoglycemia and remained prolonged during recovery to euglycemia or hyperglycemia. T-wave amplitude decreased during hypoglycemia and returned to baseline levels in both of the glycemic recovery levels. This study did not observe any ventricular or supraventricular premature beats. 

### 8.2. Spontaneous Hypoglycemia Studies and ECG

Novodvorsky et al. [82] performed spontaneous hypoglycemia studies in people with type 1 diabetes. Individuals wore Holter ECG monitors and CGMs for 96 h. Bradycardia occurred during nighttime hypoglycemia. Atrial ectopic beats were observed during daytime hypoglycemia. There were no differences in ventricular arrhythmias observed during hypoglycemia compared to euglycemia. Hypoglycemia increased QTc prolongation. 

Bachmann et al. [80] studied children under 18 years old with type 1 diabetes with CGMs and Holter ECG monitors for five nights. No arrhythmias were observed with their nocturnal hypoglycemia. QTc prolongation occurred more during hypoglycemia compared to euglycemia. Their heart rates increased during hypoglycemia. 

Stahn et al. [140] performed spontaneous hypoglycemia studies in people with type 2 diabetes treated with insulin and/or sulfonylureas and with a history of cardiovascular disease. Ventricular arrhythmias were observed at a higher rate in the individuals that experienced severe hypoglycemia.

## 9. Conclusions

Fortunately, death due to hypoglycemia is rare. However, hypoglycemia can lead to several alterations of cardiac function, mainly cardiac electrophysiology disturbances, that can lead to cardiac arrhythmias. The brain seems to be a major participant in regulating cardiac function during hypoglycemia. Both the SNS and PNS influence the heart to control heart rate, contractility, and ejection fraction. There is strong evidence for a role of the SNS in mediating cardiac arrhythmias during hypoglycemia from both clinical and animal studies. However, the recent involvement of the PNS in mediating hypoglycemia-induced arrhythmias is new and needs more research. It is intriguing that most clinical cardiac arrhythmias occur during nighttime hypoglycemia when bradycardia is also observed. This indicates a potentially unrecognized important role of the PNS in regulating cardiac arrhythmias during hypoglycemia.

## 10. Future Directions

There is still much to be discovered to gain a better understanding of how the brain regulates cardiac function during hypoglycemia. How and why both the SNS and PNS could initiate cardiac arrhythmias during hypoglycemia is unclear. If research can determine a common pathway among the two that directly causes arrhythmias, then we might be able to develop better therapeutic targets for preventing cardiovascular death from hypoglycemia in people with diabetes.

## References

[1] Cryer P.E. (2008). The Barrier of Hypoglycemia in Diabetes. Diabetes.

[2] Frier B.M. (2014). Hypoglycaemia in diabetes mellitus: Epidemiology and clinical implications. Nat. Rev. Endocrinol..

[3] Group U.K.H.S. (2007). Risk of hypoglycaemia in types 1 and 2 diabetes: Effects of treatment modalities and their duration. Diabetologia.

[4] Khunti K., Alsifri S., Aronson R., Berkovic C., Enters-Weijnen C., Forsen T., Galstyan G., Geelhoed-Duijvestijn P., Goldfracht M., Gydesen H. (2016). Rates and predictors of hypoglycaemia in 27 585 people from 24 countries with insulin-treated type 1 and type 2 diabetes: The global HAT study. Diabetes Obes. Metab..

[5] Agiostratidou G., Anhalt H., Ball D., Blonde L., Gourgari E., Harriman K.N., Kowalski A.J., Madden P., McAuliffe-Fogarty A.H., McElwee-Malloy M. (2017). Standardizing Clinically Meaningful Outcome Measures Beyond HbA(1c) for Type 1 Diabetes: A Consensus Report of the American Association of Clinical Endocrinologists, the American Association of Diabetes Educators, the American Diabetes Association, the Endocrine Society, JDRF International, The Leona M. and Harry B. Helmsley Charitable Trust, the Pediatric Endocrine Society, and the T1D Exchange. Diabetes Care.

[6] Cryer P.E. (1992). Iatrogenic hypoglycemia as a cause of hypoglycemia-associated autonomic failure in IDDM. A vicious cycle. Diabetes.

[7] Terpstra M., Moheet A., Kumar A., Eberly L.E., Seaquist E., Oz G. (2014). Changes in human brain glutamate concentration during hypoglycemia: Insights into cerebral adaptations in hypoglycemia-associated autonomic failure in type 1 diabetes. J. Cereb. Blood Flow Metab. Off. J. Int. Soc. Cereb. Blood Flow Metab..

[8] Routh V.H. (2003). Glucosensing neurons in the ventromedial hypothalamic nucleus (VMN) and hypoglycemia-associated autonomic failure (HAAF). Diabetes/Metab. Res. Rev..

[9] American Diabetes A. (2015). Glycemic Targets. Diabetes Care.

[10] Duckworth W., Abraira C., Moritz T., Reda D., Emanuele N., Reaven P.D., Zieve F.J., Marks J., Davis S.N., Hayward R. (2009). Glucose control and vascular complications in veterans with type 2 diabetes. N. Engl. J. Med..

[11] Terry T., Raravikar K., Chokrungvaranon N., Reaven P.D. (2012). Does aggressive glycemic control benefit macrovascular and microvascular disease in type 2 diabetes? Insights from ACCORD, ADVANCE, and VADT. Curr. Cardiol. Rep..

[12] Turner R.C., Holman R.R., Stratton I.M., Cull C.A., Matthews D.R., Manley S.E., Frighi V., Wright D., Neil A., Kohner E. (1998). Effect of intensive blood-glucose control with metformin on complications in overweight patients with type 2 diabetes (UKPDS 34). Lancet.

[13] Patel A., MacMahon S., Chalmers J., Neal B., Billot L., Woodward M., Marre M., Cooper M., Glasziou P., ADVANCE Collaborative Group (2008). Intensive blood glucose control and vascular outcomes in patients with type 2 diabetes. N. Engl. J. Med..

[14] Gerstein H.C., Miller M.E., Byington R.P., Goff D.C., Bigger J.T., Buse J.B., Cushman W.C., Genuth S., Ismail-Beigi F., Action to Control Cardiovascular Risk in Diabetes Study Group (2008). Effects of intensive glucose lowering in type 2 diabetes. N. Engl. J. Med..

[15] Feltbower R.G., Bodansky H.J., Patterson C.C., Parslow R.C., Stephenson C.R., Reynolds C., McKinney P.A. (2008). Acute complications and drug misuse are important causes of death for children and young adults with type 1 diabetes: Results from the Yorkshire Register of diabetes in children and young adults. Diabetes Care.

[16] Jacobson A.M., Musen G., Ryan C.M., Silvers N., Cleary P., Waberski B., Burwood A., Weinger K., Bayless M., Diabetes Control and Complications Trial/Epidemiology of Diabetes Interventions and Complications Study Research Group (2007). Long-term effect of diabetes and its treatment on cognitive function. N. Engl. J. Med..

[17] Skrivarhaug T., Bangstad H.J., Stene L.C., Sandvik L., Hanssen K.F., Joner G. (2006). Long-term mortality in a nationwide cohort of childhood-onset type 1 diabetic patients in Norway. Diabetologia.

[18] Secrest A.M., Becker D.J., Kelsey S.F., Laporte R.E., Orchard T.J. (2011). Characterizing sudden death and dead-in-bed syndrome in Type 1 diabetes: Analysis from two childhood-onset Type 1 diabetes registries. Diabet. Med. A J. Br. Diabet. Assoc..

[19] Tattersall R.B., Gill G.V. (1991). Unexplained deaths of type 1 diabetic patients. Diabet. Med..

[20] Tu E., Twigg S.M., Duflou J., Semsarian C. (2008). Causes of death in young Australians with type 1 diabetes: A review of coronial postmortem examinations. Med. J. Aust..

[21] O’Reilly M., O’Sullivan E.P., Davenport C., Smith D. (2010). “Dead in bed”: A tragic complication of type 1 diabetes mellitus. Ir. J. Med. Sci..

[22] Tanenberg R.J., Newton C.A., Drake A.J. (2010). Confirmation of Hypoglycemia in the “Dead-in-Bed” Syndrome, as Captured by a Retrospective Continuous Glucose Monitoring System. Endocr. Pract..

[23] Patel F. (1994). Diabetic death bed: Post-mortem determination of hypoglycaemia. Med. Sci. Law.

[24] Ziegler D. (1999). Cardiovascular autonomic neuropathy: Clinical manifestations and measurement. Diabetes Rev..

[25] International Hypoglycaemia Study G. (2019). Hypoglycaemia, cardiovascular disease, and mortality in diabetes: Epidemiology, pathogenesis, and management. Lancet Diabetes Endocrinol..

[26] Seaquist E.R., Anderson J., Childs B., Cryer P., Dagogo-Jack S., Fish L., Heller S.R., Rodriguez H., Rosenzweig J., Vigersky R. (2013). Hypoglycemia and diabetes: A report of a workgroup of the American Diabetes Association and the Endocrine Society. J. Clin. Endocrinol. Metab..

[27] Bisgaard Bengtsen M., Moller N. (2021). Mini-review: Glucagon responses in type 1 diabetes—A matter of complexity. Physiol. Rep..

[28] Lopez-Gambero A.J., Martinez F., Salazar K., Cifuentes M., Nualart F. (2019). Brain Glucose-Sensing Mechanism and Energy Homeostasis. Mol. Neurobiol..

[29] Coll A.P., Yeo G.S. (2013). The hypothalamus and metabolism: Integrating signals to control energy and glucose homeostasis. Curr. Opin. Pharmacol..

[30] Schwartz M.W., Figlewicz D.P., Baskin D.G., Woods S.C., Porte D. (1992). Insulin in the brain: A hormonal regulator of energy balance. Endocr. Rev..

[31] Robinson R.T., Harris N.D., Ireland R.H., Lee S., Newman C., Heller S.R. (2003). Mechanisms of abnormal cardiac repolarization during insulin-induced hypoglycemia. Diabetes.

[32] Chow E., Bernjak A., Walkinshaw E., Lubina-Solomon A., Freeman J., Macdonald I.A., Sheridan P.J., Heller S.R. (2017). Cardiac Autonomic Regulation and Repolarization During Acute Experimental Hypoglycemia in Type 2 Diabetes. Diabetes.

[33] Donovan C.M., Watts A.G. (2014). Peripheral and Central Glucose Sensing In Hypoglycemic Detection. Physiology.

[34] Watts A.G., Donovan C.M. (2010). Sweet talk in the brain: Glucosensing, neural networks, and hypoglycemic counterregulation. Front. Neuroendocr..

[35] Dunn-Meynell A.A., Routh V.H., Kang L., Gaspers L., Levin B.E. (2002). Glucokinase is the likely mediator of glucosensing in both glucose-excited and glucose-inhibited central neurons. Diabetes.

[36] Guan H.Z., Dong J., Jiang Z.Y., Chen X. (2017). alpha-MSH Influences the Excitability of Feeding-Related Neurons in the Hypothalamus and Dorsal Vagal Complex of Rats. Biomed. Res. Int..

[37] Wang R., Liu X., Hentges S.T., Dunn-Meynell A.A., Levin B.E., Wang W., Routh V.H. (2004). The regulation of glucose-excited neurons in the hypothalamic arcuate nucleus by glucose and feeding-relevant peptides. Diabetes.

[38] Katafuchi T., Oomura Y., Yoshimatsu H. (1985). Single Neuron Activity in the Rat Lateral Hypothalamus during 2-Deoxy-D-Glucose Induced and Natural Feeding-Behavior. Brain Res..

[39] Zhu J.N., Guo C.L., Li H.Z., Wang J.J. (2007). Dorsomedial hypothalamic nucleus neurons integrate important peripheral feeding-related signals in rats. J. Neurosci. Res..

[40] Funahashi M., Adachi A. (1993). Glucose-Responsive Neurons Exist within the Area Postrema of the Rat—In-Vitro Study on the Isolated Slice Preparation. Brain Res. Bull..

[41] Mimee A., Ferguson A.V. (2015). Glycemic state regulates melanocortin, but not nesfatin-1, responsiveness of glucose-sensing neurons in the nucleus of the solitary tract. Am. J. Physiol.-Regul. Integr. Comp. Physiol..

[42] Flak J.N., Goforth P.B., Dell’Orco J., Sabatini P.V., Li C.E., Bozadjieva N., Sorensen M., Valenta A., Rupp A., Affinati A.H. (2020). Ventromedial hypothalamic nucleus neuronal subset regulates blood glucose independently of insulin. J. Clin. Investig..

[43] Meek T.H., Nelson J.T., Matsen M.E., Dorfman M.D., Guyenet S.J., Damian V., Allison M.B., Scarlett J.M., Nguyen H.T., Thaler J.P. (2016). Functional identification of a neurocircuit regulating blood glucose. Proc. Natl. Acad. Sci. USA.

[44] Faber C.L., Matsen M.E., Velasco K.R., Damian V., Phan B.A., Adam D., Therattil A., Schwartz M.W., Morton G.J. (2018). Distinct Neuronal Projections From the Hypothalamic Ventromedial Nucleus Mediate Glycemic and Behavioral Effects. Diabetes.

[45] Kang L., Routh V.H., Kuzhikandathil E.V., Gaspers L.D., Levin B.E. (2004). Physiological and molecular characteristics of rat hypothalamic ventromedial nucleus glucosensing neurons. Diabetes.

[46] Routh V.H. (2010). Glucose Sensing Neurons in the Ventromedial Hypothalamus. Sensors.

[47] McCrimmon R.J., Shaw M., Fan X.N., Cheng H.Y., Ding Y.Y., Vella M.C., Zhou L.G., Mcnay E.C., Sherwin R.S. (2008). Key role for AMP-activated protein kinase in the ventromedial hypothalamus in regulating counterregulatory hormone responses to acute hypoglycemia. Diabetes.

[48] Fioramonti X., Marsollier N., Song Z.T., Fakira K.A., Patel R.M., Brown S., Duparc T., Pica-Mendez A., Sanders N.M., Knauf C. (2010). Ventromedial Hypothalamic Nitric Oxide Production Is Necessary for Hypoglycemia Detection and Counterregulation. Diabetes.

[49] Amiel S.A., Simonson D.C., Tamborlane W.V., Defronzo R.A., Sherwin R.S. (1987). Rate of Glucose Fall Does Not Affect Counterregulatory Hormone Responses to Hypoglycemia in Normal and Diabetic Humans. Diabetes.

[50] Frizzell R.T., Jones E.M., Davis S.N., Biggers D.W., Myers S.R., Connolly C.C., Neal D.W., Jaspan J.B., Cherrington A.D. (1993). Counterregulation during Hypoglycemia Is Directed by Widespread Brain-Regions. Diabetes.

[51] Musen G., Simonson D.C., Bolo N.R., Driscoll A., Weinger K., Raji A., Theberge J., Renshaw P.F., Jacobson A.M. (2008). Regional brain activation during hypoglycemia in type 1 diabetes. J. Clin. Endocr. Metab..

[52] Medeiros N., Dai L., Ferguson A.V. (2012). Glucose-responsive neurons in the subfornical organ of the rat--a novel site for direct CNS monitoring of circulating glucose. Neuroscience.

[53] Dallaporta M., Himmi T., Perrin J., Orsini J.C. (1999). Solitary tract nucleus sensitivity to moderate changes in glucose level. Neuroreport.

[54] Shapiro R.E., Miselis R.R. (1985). The Central Neural Connections of the Area Postrema of the Rat. J. Comp. Neurol..

[55] Martinez F., Cifuentes M., Tapia J.C., Nualart F. (2019). The median eminence as the hypothalamic area involved in rapid transfer of glucose to the brain: Functional and cellular mechanisms. J. Mol. Med..

[56] Kohnke S., Buller S., Nuzzaci D., Ridley K., Lam B., Pivonkova H., Bentsen M.A., Alonge K.M., Zhao C., Tadross J. (2021). Nutritional regulation of oligodendrocyte differentiation regulates perineuronal net remodeling in the median eminence. Cell Rep..

[57] Lynch R.M., Tompkins L.S., Brooks H.L., Dunn-Meynell A.A., Levin B.E. (2000). Localization of glucokinase gene expression in the rat brain. Diabetes.

[58] Boychuk C.R., Gyarmati P., Xu H., Smith B.N. (2015). Glucose sensing by GABAergic neurons in the mouse nucleus tractus solitarii. J. Neurophysiol..

[59] Lamy C.M., Sanno H., Labouebe G., Picard A., Magnan C., Chatton J.Y., Thorens B. (2014). Hypoglycemia-activated GLUT2 neurons of the nucleus tractus solitarius stimulate vagal activity and glucagon secretion. Cell Metab..

[60] Loewy A., Spyer K., Loewy A., Spyer K.M. (1990). Vagal preganglionic neurons. Central Regulation of Autonomic Functions.

[61] Frangos E., Komisaruk B.R. (2017). Access to Vagal Projections via Cutaneous Electrical Stimulation of the Neck: fMRI Evidence in Healthy Humans. Brain Stimul..

[62] Buller K., Xu Y., Dayas C., Day T. (2001). Dorsal and ventral medullary catecholamine cell groups contribute differentially to systemic interleukin-1beta-induced hypothalamic pituitary adrenal axis responses. Neuroendocrinology.

[63] Palma J.A., Benarroch E.E. (2014). Neural control of the heart: Recent concepts and clinical correlations. Neurology.

[64] Thayer J.F., Lane R.D. (2009). Claude Bernard and the heart-brain connection: Further elaboration of a model of neurovisceral integration. Neurosci. Biobehav. Rev..

[65] Ahern G.L., Sollers J.J., Lane R.D., Labiner D.M., Herring A.M., Weinand M.E., Hutzler R., Thayer J.F. (2001). Heart rate and heart rate variability changes in the intracarotid sodium amobarbital test. Epilepsia.

[66] Teves D., Videen T.O., Cryer P.E., Powers W.J. (2004). Activation of human medial prefrontal cortex during autonomic responses to hypoglycemia. Proc. Natl. Acad. Sci. USA.

[67] Hwang J.J., Parikh L., Lacadie C., Seo D., Lam W., Hamza M., Schmidt C., Dai F., Sejling A.S., Belfort-DeAguiar R. (2018). Hypoglycemia unawareness in type 1 diabetes suppresses brain responses to hypoglycemia. J. Clin. Investig..

[68] Saha S. (2005). Role of the central nucleus of the amygdala in the control of blood pressure: Descending pathways to medullary cardiovascular nuclei. Clin. Exp. Pharmacol. Physiol..

[69] Evans S.B., Wilkinson C.W., Gronbeck P., Bennett J.L., Taborsky G.J., Figlewicz D.P. (2003). Inactivation of the PVN during hypoglycemia partially simulates hypoglycemia-associated autonomic failure. Am. J. Physiol.-Regul. Integr. Comp. Physiol..

[70] Borg W.P., During M.J., Sherwin R.S., Borg M.A., Brines M.L., Shulman G.I. (1994). Ventromedial Hypothalamic-Lesions in Rats Suppress Counterregulatory Responses to Hypoglycemia. J. Clin. Investig..

[71] Puskas N., Papp R.S., Gallatz K., Palkovits M. (2010). Interactions between orexin-immunoreactive fibers and adrenaline or noradrenaline-expressing neurons of the lower brainstem in rats and mice. Peptides.

[72] Melville K.I., Blum B., Shister H.E., Silver M.D. (1963). Cardiac Ischemic Changes and Arrhythmias Induced by Hypothalamic Stimulation. Am. J. Cardiol..

[73] Paranjape S.A., Briski K.P. (2005). Recurrent insulin-induced hypoglycemia causes site-specific patterns of habituation or amplification of CNS neuronal genomic activation. Neuroscience.

[74] Diggs-Andrews K.A., Zhang X., Song Z., Daphna-Iken D., Routh V.H., Fisher S.J. (2010). Brain insulin action regulates hypothalamic glucose sensing and the counterregulatory response to hypoglycemia. Diabetes.

[75] Gordan R., Gwathmey J.K., Xie L.H. (2015). Autonomic and endocrine control of cardiovascular function. World J. Cardiol..

[76] Seguela P., Wadiche J., Dineley-Miller K., Dani J.A., Patrick J.W. (1993). Molecular cloning, functional properties, and distribution of rat brain alpha 7: A nicotinic cation channel highly permeable to calcium. J. Neurosci. Off. J. Soc. Neurosci..

[77] Wada E., Wada K., Boulter J., Deneris E., Heinemann S., Patrick J., Swanson L.W. (1989). Distribution of alpha 2, alpha 3, alpha 4, and beta 2 neuronal nicotinic receptor subunit mRNAs in the central nervous system: A hybridization histochemical study in the rat. J. Comp. Neurol..

[78] Carlson A.B., Kraus G.P. (2023). Physiology, Cholinergic Receptors. StatPearls.

[79] Colomer C., Olivos-Ore L.A., Vincent A., McIntosh J.M., Artalejo A.R., Guerineau N.C. (2010). Functional characterization of alpha9-containing cholinergic nicotinic receptors in the rat adrenal medulla: Implication in stress-induced functional plasticity. J. Neurosci. Off. J. Soc. Neurosci..

[80] Bachmann S., Auderset A., Burckhardt M.A., Szinnai G., Hess M., Zumsteg U., Denhaerynck K., Donner B. (2021). Autonomic cardiac regulation during spontaneous nocturnal hypoglycemia in children with type 1 diabetes. Pediatr. Diabetes.

[81] Chow E., Bernjak A., Williams S., Fawdry R.A., Hibbert S., Freeman J., Sheridan P.J., Heller S.R. (2014). Risk of cardiac arrhythmnias during hypoglycemia in patients with type 2 diabetes and cardiovascular risk. Diabetes.

[82] Novodvorsky P., Bernjak A., Chow E., Iqbal A., Sellors L., Williams S., Fawdry R.A., Parekh B., Jacques R.M., Marques J.L.B. (2017). Diurnal Differences in Risk of Cardiac Arrhythmias During Spontaneous Hypoglycemia in Young People With Type 1 Diabetes. Diabetes Care.

[83] Ahmet A., Dagenais S., Barrowman N.J., Collins C.J., Lawson M.L. (2011). Prevalence of nocturnal hypoglycemia in pediatric type 1 diabetes: A pilot study using continuous glucose monitoring. J. Pediatr..

[84] Wiltshire E.J., Newton K., McTavish L. (2006). Unrecognised hypoglycaemia in children and adolescents with type 1 diabetes using the continuous glucose monitoring system: Prevalence and contributors. J. Paediatr. Child Health.

[85] Gordon S.G., Kittleson M.D., Maddison J.E., Page S.W., Church D.B. (2008). Drugs used in the management of heart disease and cardiac arrhythmias. Small Animal Clinical Pharmacology.

[86] Reno C.M., Bayles J., Huang Y., Oxspring M., Hirahara A.M., Dosdall D.J., Fisher S.J. (2019). Severe Hypoglycemia-Induced Fatal Cardiac Arrhythmias Are Mediated by the Parasympathetic Nervous System in Rats. Diabetes.

[87] Reno C.M., Daphna-Iken D., Chen Y.S., Vanderweele J., Jethi K., Fisher S.J. (2013). Severe hypoglycemia-induced lethal cardiac arrhythmias are mediated by sympathoadrenal activation. Diabetes.

[88] Reno C.M., VanderWeele J., Bayles J., Litvin M., Skinner A., Jordan A., Daphna-Iken D., Fisher S.J. (2017). Severe Hypoglycemia-Induced Fatal Cardiac Arrhythmias are Augmented by Diabetes and Attenuated by Recurrent Hypoglycemia. Diabetes.

[89] Gill G., Woodward A., Casson I., Weston P. (2009). Cardiac arrhythmia and nocturnal hypoglycaemia in type 1 diabetes-the ‘dead in bed’ syndrome revisited. Diabetologia.

[90] Marques J.L., George E., Peacey S.R., Harris N.D., Macdonald I.A., Cochrane T., Heller S.R. (1997). Altered ventricular repolarization during hypoglycaemia in patients with diabetes. Diabet. Med..

[91] Landstedt-Hallin L., Englund A., Adamson U., Lins P.E. (1999). Increased QT dispersion during hypoglycaemia in patients with type 2 diabetes mellitus. J. Intern. Med..

[92] Kubiak T., Wittig A., Koll C., Mraz B., Gustav J., Herrmann U., Weber H., Kerner W. (2010). Continuous glucose monitoring reveals associations of glucose levels with QT interval length. Diabetes Technol. Ther..

[93] Christensen T.F., Cichosz S.L., Tarnow L., Randlov J., Kristensen L.E., Struijk J.J., Eldrup E., Hejlesen O.K. (2014). Hypoglycaemia and QT interval prolongation in type 1 diabetes—Bridging the gap between clamp studies and spontaneous episodes. J. Diabetes Complicat..

[94] Mylona M., Liatis S., Anastasiadis G., Kapelios C., Kokkinos A. (2020). Severe iatrogenic hypoglycaemia requiring medical assistance is associated with concurrent prolongation of the QTc interval. Diabetes Res. Clin. Pract..

[95] Cox A.J., Azeem A., Yeboah J., Soliman E.Z., Aggarwal S.R., Bertoni A.G., Carr J.J., Freedman B.I., Herrington D.M., Bowden D.W. (2014). Heart Rate- Corrected QT Interval Is an Independent Predictor of AllCause and Cardiovascular Mortality in Individuals WithType 2 Diabetes: The Diabetes Heart Study. Diabetes Care.

[96] Rossing P., Breum L., Major-Pedersen A., Sato A., Winding H., Pietersen A., Kastrup J., Parving H.H. (2001). Prolonged QTc interval predicts mortality in patients with Type 1 diabetes mellitus. Diabet. Med..

[97] Frier B.M., Schernthaner G., Heller S.R. (2011). Hypoglycemia and cardiovascular risks. Diabetes Care.

[98] Laitinen T., Lyyra-Laitinen T., Huopio H., Vauhkonen I., Halonen T., Hartikainen J., Niskanen L., Laakso M. (2008). Electrocardiographic alterations during hyperinsulinemic hypoglycemia in healthy subjects. Ann. Noninvasive Electrocardiol. Off. J. Int. Soc. Holter Noninvasive Electrocardiol. Inc..

[99] Soydan N., Bretzel R.G., Fischer B., Wagenlehner F., Pilatz A., Linn T. (2013). Reduced capacity of heart rate regulation in response to mild hypoglycemia induced by glibenclamide and physical exercise in type 2 diabetes. Metab. Clin. Exp..

[100] Andersen A., Bagger J.I., Baldassarre M.P.A., Christensen M.B., Abelin K.U., Faber J., Pedersen-Bjergaard U., Holst J.J., Lindhardt T.B., Gislason G. (2021). Acute hypoglycemia and risk of cardiac arrhythmias in insulin-treated type 2 diabetes and controls. Eur. J. Endocrinol..

[101] Andersen A., Bagger J.I., Sorensen S.K., Baldassarre M.P.A., Pedersen-Bjergaard U., Forman J.L., Gislason G., Lindhardt T.B., Knop F.K., Vilsboll T. (2021). Associations of hypoglycemia, glycemic variability and risk of cardiac arrhythmias in insulin-treated patients with type 2 diabetes: A prospective, observational study. Cardiovasc. Diabetol..

[102] Pollock G., Brady W.J., Hargarten S., DeSilvey D., Carner C.T. (1996). Hypoglycemia manifested by sinus bradycardia: A report of three cases. Acad. Emerg. Med..

[103] Bolognesi R., Tsialtas D., Bolognesi M.G., Giumelli C. (2011). Marked sinus bradycardia and QT prolongation in a diabetic patient with severe hypoglycemia. J. Diabetes Complicat..

[104] Nordin C. (2014). The proarrhythmic effect of hypoglycemia: Evidence for increased risk from ischemia and bradycardia. Acta Diabetol..

[105] Reno C.M., Skinner A., Bayles J., Chen Y.S., Daphna-Iken D., Fisher S.J. (2018). Severe hypoglycemia-induced sudden death is mediated by both cardiac arrhythmias and seizures. Am. J. Physiol. Endocrinol. Metab..

[106] Greenhoot J.H., Reichenbach D.D. (1969). Cardiac injury and subarachnoid hemorrhage. A clinical, pathological, and physiological correlation. J. Neurosurg..

[107] Reno C.M., Bayles J., Skinner A., Fisher S.J. (2018). Glibenclamide Prevents Hypoglycemia-Induced Fatal Cardiac Arrhythmias in Rats. Endocrinology.

[108] Cardoso R., Graffunder F.P., Ternes C.M.P., Fernandes A., Rocha A.V., Fernandes G., Bhatt D.L. (2021). SGLT2 inhibitors decrease cardiovascular death and heart failure hospitalizations in patients with heart failure: A systematic review and meta-analysis. EClinicalMedicine.

[109] Zinman B., Wanner C., Lachin J.M., Fitchett D., Bluhmki E., Hantel S., Mattheus M., Devins T., Johansen O.E., Woerle H.J. (2015). Empagliflozin, Cardiovascular Outcomes, and Mortality in Type 2 Diabetes. N. Engl. J. Med..

[110] Fawzy A.M., Rivera-Caravaca J.M., Underhill P., Fauchier L., Lip G.Y.H. (2023). Incident heart failure, arrhythmias and cardiovascular outcomes with sodium-glucose cotransporter 2 (SGLT2) inhibitor use in patients with diabetes: Insights from a global federated electronic medical record database. Diabetes Obes. Metab..

[111] Yang F., Meng R., Zhu D.L. (2020). Cardiovascular effects and mechanisms of sodium-glucose cotransporter-2 inhibitors. Chronic Dis. Transl. Med..

[112] Cahill G.F. (2006). Fuel metabolism in starvation. Annu. Rev. Nutr..

[113] Ferrannini E., Mark M., Mayoux E. (2016). CV Protection in the EMPA-REG OUTCOME Trial: A “Thrifty Substrate” Hypothesis. Diabetes Care.

[114] Dobrev D., Wehrens X.H. (2014). Role of RyR2 phosphorylation in heart failure and arrhythmias: Controversies around ryanodine receptor phosphorylation in cardiac disease. Circ. Res..

[115] Lehnart S.E., Terrenoire C., Reiken S., Wehrens X.H., Song L.S., Tillman E.J., Mancarella S., Coromilas J., Lederer W.J., Kass R.S. (2006). Stabilization of cardiac ryanodine receptor prevents intracellular calcium leak and arrhythmias. Proc. Natl. Acad. Sci. USA.

[116] Shan J., Betzenhauser M.J., Kushnir A., Reiken S., Meli A.C., Wronska A., Dura M., Chen B.X., Marks A.R. (2010). Role of chronic ryanodine receptor phosphorylation in heart failure and beta-adrenergic receptor blockade in mice. J. Clin. Investig..

[117] Ho H.T., Belevych A.E., Liu B., Bonilla I.M., Radwanski P.B., Kubasov I.V., Valdivia H.H., Schober K., Carnes C.A., Gyorke S. (2016). Muscarinic Stimulation Facilitates Sarcoplasmic Reticulum Ca Release by Modulating Ryanodine Receptor 2 Phosphorylation Through Protein Kinase G and Ca/Calmodulin-Dependent Protein Kinase II. Hypertension.

[118] Baine S., Thomas J., Bonilla I., Ivanova M., Belevych A., Li J., Veeraraghavan R., Radwanski P.B., Carnes C., Gyorke S. (2020). Muscarinic-dependent phosphorylation of the cardiac ryanodine receptor by protein kinase G is mediated by PI3K-AKT-nNOS signaling. J. Biol. Chem..

[119] Bround M.J., Asghari P., Wambolt R.B., Bohunek L., Smits C., Philit M., Kieffer T.J., Lakatta E.G., Boheler K.R., Moore E.D.W. (2012). Cardiac ryanodine receptors control heart rate and rhythmicity in adult mice. Cardiovasc. Res..

[120] Fisher B.M., Gillen G., Hepburn D.A., Dargie H.J., Frier B.M. (1990). Cardiac responses to acute insulin-induced hypoglycemia in humans. Am. J. Physiol..

[121] Hilsted J., Bonde-Petersen F., Norgaard M.B., Greniman M., Christensen N.J., Parving H.H., Suzuki M. (1984). Haemodynamic changes in insulin-induced hypoglycaemia in normal man. Diabetologia.

[122] Yakubovich N., Gerstein H.C. (2011). Serious cardiovascular outcomes in diabetes: The role of hypoglycemia. Circulation.

[123] Hsu P.F., Sung S.H., Cheng H.M., Yeh J.S., Liu W.L., Chan W.L., Chen C.H., Chou P., Chuang S.Y. (2013). Association of clinical symptomatic hypoglycemia with cardiovascular events and total mortality in type 2 diabetes: A nationwide population-based study. Diabetes Care.

[124] Desouza C.V., Bolli G.B., Fonseca V. (2010). Hypoglycemia, diabetes, and cardiovascular events. Diabetes Care.

[125] Lohmann F.W., Loesment W.A., Kaehler H. (1990). Beta-receptor blockade, physical activity, and metabolism. J. Cardiovasc. Pharmacol..

[126] Wright R.J., Frier B.M. (2008). Vascular disease and diabetes: Is hypoglycaemia an aggravating factor?. Diabetes-Metab. Res..

[127] Gogitidze Joy N., Hedrington M.S., Briscoe V.J., Tate D.B., Ertl A.C., Davis S.N. (2010). Effects of acute hypoglycemia on inflammatory and pro-atherothrombotic biomarkers in individuals with type 1 diabetes and healthy individuals. Diabetes Care.

[128] Iqbal A., Prince L.R., Novodvorsky P., Bernjak A., Thomas M.R., Birch L., Lambert D., Kay L.J., Wright F.J., Macdonald I.A. (2019). Effect of Hypoglycemia on Inflammatory Responses and the Response to Low-Dose Endotoxemia in Humans. J. Clin. Endocrinol. Metab..

[129] Fisher B.M., Quin J.D., Rumley A., Lennie S.E., Small M., MacCuish A.C., Lowe G.D. (1991). Effects of acute insulin-induced hypoglycaemia on haemostasis, fibrinolysis and haemorheology in insulin-dependent diabetic patients and control subjects. Clin. Sci..

[130] Chow E., Iqbal A., Walkinshaw E., Phoenix F., Macdonald I.A., Storey R.F., Ajjan R., Heller S.R. (2018). Prolonged Prothrombotic Effects of Antecedent Hypoglycemia in Individuals With Type 2 Diabetes. Diabetes Care.

[131] Verhulst C.E.M., van Heck J.I.P., Fabricius T.W., Stienstra R., Teerenstra S., McCrimmon R.J., Tack C.J., Pedersen-Bjergaard U., de Galan B.E. (2022). Sustained Proinflammatory Effects of Hypoglycemia in People With Type 2 Diabetes and in People Without Diabetes. Diabetes.

[132] Ceriello A., Novials A., Ortega E., La Sala L., Pujadas G., Testa R., Bonfigli A.R., Esposito K., Giugliano D. (2012). Evidence that hyperglycemia after recovery from hypoglycemia worsens endothelial function and increases oxidative stress and inflammation in healthy control subjects and subjects with type 1 diabetes. Diabetes.

[133] Wright R.J., Newby D.E., Stirling D., Ludlam C.A., Macdonald I.A., Frier B.M. (2010). Effects of acute insulin-induced hypoglycemia on indices of inflammation: Putative mechanism for aggravating vascular disease in diabetes. Diabetes Care.

[134] Dagher Z., Ruderman N., Tornheim K., Ido Y. (2001). Acute regulation of fatty acid oxidation and amp-activated protein kinase in human umbilical vein endothelial cells. Circ. Res..

[135] Razavi Nematollahi L., Kitabchi A.E., Stentz F.B., Wan J.Y., Larijani B.A., Tehrani M.M., Gozashti M.H., Omidfar K., Taheri E. (2009). Proinflammatory cytokines in response to insulin-induced hypoglycemic stress in healthy subjects. Metab. Clin. Exp..

[136] Cardoso S., Santos R.X., Correia S.C., Carvalho C., Santos M.S., Baldeiras I., Oliveira C.R., Moreira P.I. (2013). Insulin-induced recurrent hypoglycemia exacerbates diabetic brain mitochondrial dysfunction and oxidative imbalance. Neurobiol. Dis..

[137] An D., Rodrigues B. (2006). Role of changes in cardiac metabolism in development of diabetic cardiomyopathy. Am. J. Physiology. Heart Circ. Physiol..

[138] Reno-Bernstein C.M., Oxspring M., Bayles J., Huang E.Y., Holiday I., Fisher S.J. (2022). Vitamin E treatment in insulin-deficient diabetic rats reduces cardiac arrhythmias and mortality during severe hypoglycemia. Am. J. Physiol. Endocrinol. Metab..

[139] Andreasen C.R., Andersen A., Hagelqvist P.G., Maytham K., Lauritsen J.V., Engberg S., Faber J., Pedersen-Bjergaard U., Knop F.K., Vilsboll T. (2023). Sustained heart rate-corrected QT prolongation during recovery from hypoglycaemia in people with type 1 diabetes, independently of recovery to hyperglycaemia or euglycaemia. Diabetes Obes. Metab..

[140] Stahn A., Pistrosch F., Ganz X., Teige M., Koehler C., Bornstein S., Hanefeld M. (2014). Relationship between hypoglycemic episodes and ventricular arrhythmias in patients with type 2 diabetes and cardiovascular diseases: Silent hypoglycemias and silent arrhythmias. Diabetes Care.

